# Air quality in Mexico City during the COVID-19 lockdown possibly decreased COPD exacerbations

**DOI:** 10.1183/23120541.00183-2022

**Published:** 2022-10-10

**Authors:** Francisco Montiel-Lopez, Daniela Rodríguez-Ramírez, Maricruz Cassou-Martínez, María Cristina Miranda-Márquez, Claudia González-González, Raúl H. Sansores, María Eugenia Mayar-Maya, Rogelio Pérez-Padilla, Robinson Emmanuel Robles-Hernández, Ireri Thirion-Romero, Oliver Pérez-Bautista, Alejandra Ramírez-Venegas

**Affiliations:** 1 Dept of Tobacco Smoking and COPD Research, Instituto Nacional de Enfermedades Respiratorias Ismael Cosío Villegas, Mexico City, Mexico; 2 Economy Faculty, Universidad Autónoma Benito Juárez de Oaxaca, Oaxaca, Mexico; 3 Dept of Respiratory Medicine, Medica Sur Clinic and Foundation, Mexico City, Mexico

## Abstract

During the first year of the COVID-19 pandemic, various authors reported that acute exacerbations of COPD (AECOPD) were reduced [1–4]. In Mexico City, during the beginning of the pandemic, a voluntary lockdown was declared and therefore the adherence to health measures was more flexible and less stringent in the general population than in other countries [5]. However, because there was a reduction of exposure to air pollutants such as particulate matter with a diameter <10 μm (PM_10_) and <2.5 µm (PM_2.5_), as well as total suspended particles levels during the first year of the pandemic, especially among patients living in urban areas like Mexico City, AECOPD could be diminished as other countries have reported [1–4].

## Abstract 2

**The reduction of air pollution during the #COVID19 lockdown in Mexico City possibly reduced the exacerbation rate in #COPD patients due to biomass and tobacco despite that the self-isolation was not as strict as expected.** https://bit.ly/3Iyv98t

*To the Editor:*

During the first year of the COVID-19 pandemic, various authors reported that acute exacerbations of COPD (AECOPD) were reduced [1–4]. In Mexico City, during the beginning of the pandemic, a voluntary lockdown was declared and therefore the adherence to health measures was more flexible and less stringent in the general population than in other countries [5]. However, because there was a reduction of exposure to air pollutants such as particulate matter with a diameter <10 μm (PM_10_) and <2.5 µm (PM_2.5_), as well as total suspended particles levels during the first year of the pandemic, especially among patients living in urban areas like Mexico City, AECOPD could be diminished as other countries have reported [1–4]. In rural areas of Mexico, because of the lack of access to clean electricity and gas, many women in these communities cook their food by burning firewood, exposing themselves to biomass smoke almost all day throughout their lives, making them more susceptible to developing COPD. COPD exacerbations due to biomass in rural areas during the COVID-19 pandemic should not decrease because the environmental pollutants did not change. The primary objective of this study was to investigate if there was a reduction in AECOPD during the first year of the COVID-19 pandemic in comparison with the previous year. Because women with biomass exposure represent an important group of people living with COPD in Mexico [6], a secondary objective was to investigate the difference in AECOPD between the COPD due to biomass exposure (BE-COPD) and the COPD due to tobacco smoking (TE-COPD) groups.

This was a cross-sectional, retrospective study made through a telephone survey to BE-COPD and TE-COPD patients during the first year of the COVID-19 pandemic, and a review of patients’ records of a subcohort of BE-COPD and TE-COPD patients from the COPD clinic at the National Institute of Respiratory Diseases in Mexico City. The study had two phases. The first consisted of a telephone survey of patients from March 2020 to February 2021 (referred to as 2020–2021 hereon), and the second phase consisted of a review of AECOPD from March 2019 to February 2020 (referred to as 2019–2020 hereon) from the medical records of the 210 patients that answered the survey. The surveys focused on the presence of moderate to severe COPD exacerbations during these periods. In order to establish a relationship between exacerbations and adherence to preventive health measures, patients were asked about their compliance with social distancing and self-isolation, use of face masks, and handwashing during the first year of the pandemic [7]. In order to investigate whether the levels of pollutants had changed between 2019–2020 and 2020–2021, public records of suspended particles in Mexico from 2019 to 2021 were collected and analysed [8].

Logistic regression analysis was performed to evaluate risk factors associated with the presence of AECOPD. A negative binomial model used to compare COPD exacerbation rates per year adjusted by sex, COPD group and age. Stata 14.0 was used for statistical analysis.

Table 1 shows the difference between BE-COPD and TE-COPD in demographics and exacerbations characteristics. For this study, 75 (36%) patients belonged to the BE-COPD group and 135 (64%) belonged to the TE-COPD group. A higher proportion of BE-COPD patients lived in rural areas than TE-COPD patients (39% *versus* 6%, p<0.001) and had a lower socioeconomic level (p<0.001).

**TABLE 1:** Sociodemographic and exacerbation characteristics by COPD groups

**Variables**	**BE-COPD**	**TE-COPD**	**Total**	**p-value**
**Patients n**	75	135	210	
**Age, years**	76±11	73±9	74±10	0.004
**Female**	67 (89)	48 (36)	115 (55)	<0.001
**Rural residence**	29 (39)	8 (6)	37 (18)	<0.001
**Low socioeconomic status**	67 (89)	83 (62)	150 (71)	<0.001
**Low self-reported adherence to preventive measures**	28 (38)	41 (31)	69 (33)	0.272
**Strict indoor isolation**	36 (30)	28 (45)	64 (35)	0.035
**FEV_1_ % of predicted**	70±22	57±22	62±23	<0.001
**2019–2020 patients with exacerbations**	19 (25)	37 (27)	56 (27)	0.745
**2020–2021 patients with exacerbations**	13 (17)	23 (17)	36 (17)	0.956

Data presented as mean±sd or n (%), unless otherwise stated. BE-COPD: COPD due to exposure to biomass smoke; TE-COPD: COPD due to exposure to tobacco smoke; FEV_1_: forced expiratory volume in the first second.

When evaluating the adherence of preventive health measures against COVID-19 contagion, including social distancing, handwashing and the use of facemasks, 33% of all patients reported low adherence to these measures without a significant statistical difference between COPD groups. Regarding self-isolation, when asking if patients remained indoors between 2020 and 2021, there was a higher proportion of TE-COPD patients (45%) that stayed inside in comparison to the BE-COPD group (30%) (p=0.035). However, it is noticeable that 65% of all patients answered that they did not stay at home most of the time during this period. Regarding the number of patients that presented an AECOPD in 2019–2020, 27% of them reported at least one moderate exacerbation whereas in 2020–2021, 17% of them did, without significant difference between COPD groups. The AECOPD rate in 2019–2020 was 0.47 (95% CI 0.06–3.41) and in 2020–2021 was 0.18 (95% CI 0.02–1.32, p<0.001); results were adjusted by COPD group, sex and age. There were no significant differences in the reduction of exacerbation rate nor the severity between BE-COPD and TE-COPD patients. There was no relationship between remaining indoors during the first year of the pandemic and AECOPD incidence in the total population and BE- and TE-COPD groups. When we analysed COPD group, place of residence, socioeconomic status, health measurements adherence (social distancing, handwashing and use of facemasks) and self-isolation as risk factors for exacerbations during 2020–2021, the logistic regression analysis did not show an association.

This study demonstrated a significant reduction in the number AECOPD from March 2020 to February 2021 in comparison with the same period in 2019–2020, without differences between BE-COPD and TE-COPD.

As other authors have reported, we consider that lockdown measures for COPD patients during the pandemic period should be a determinant factor in reducing exacerbations. Nevertheless, there was no association between remaining indoors during the first year of the pandemic and AECOPD. Self-isolation was not very strict and was insufficient to decrease patients’ risk for developing exacerbations. However, the decrease of environmental pollution in an urban area like Mexico City could be one of the most determinant factors that may contribute to the decrease of AECOPD in 2020. In Mexico City, there was a significant decrease in environmental pollution during in 2020 compared with the previous year in PM_10_, PM_2.5_ and total suspended particle levels (45±21 ppm *versus* 40±21 ppm, p=0017; 20±10 ppm *versus* 16±7 ppm, p<0.001; 99±43 ppm *versus* 89±37 ppm, p=0.046 respectively) [8]. The study was unable to analyse a relationship between levels of pollutants and exacerbations. We can only point out that the time in which the levels of pollutants were measured were similar when we conducted the survey of BE-COPD and TE-COPD patients.

In urban areas in the USA, during the COVID-19 pandemic, the PM_2.5_ and NO_2_ levels decreased 25% during 2020 in comparison with 2017–2019 [9]. In China, during the pandemic, a reduction in NO_2_ and PM_2.5_ has been attributed to reduced mortality including cardiovascular and pulmonary causes [10, 11]. Because environmental pollution has been demonstrated to increase respiratory symptoms [12] and mortality [12, 13], the reduction of these particles in Mexico City also produced a favourable impact on the respiratory health of COPD patients. The BE-COPD group showed a similar reduction of AECOPD as the TE-COPD group. Despite that BE-COPD patients suffered from greater socioeconomic disadvantages and lower measurements of isolation during COVID-19 pandemic, the fact that 60% of them lived in Mexico's metropolitan area, they could have benefited in their AECOPD rate similarly to TE-COPD by the reduction of environmental pollution. The absence of a statistical difference in reduction of exacerbation rate between the two groups may be due to small sample size.

The improvement of the air quality in Mexico City during the COVID-19 lockdown, as other countries with high levels of air pollution reported, may contribute to the reduction of AECOPD equally in BE-COPD and TE-COPD groups.
